# Allergen-induced anxiety-like behavior is associated with disruption of medial prefrontal cortex - amygdala circuit

**DOI:** 10.1038/s41598-019-55539-3

**Published:** 2019-12-20

**Authors:** Kolsoum Dehdar, Shirin Mahdidoust, Morteza Salimi, Leila Gholami-Mahtaj, Milad Nazari, Sadeq Mohammadi, Samaneh Dehghan, Hamidreza Jamaati, Reza Khosrowabadi, Abbas Nasiraei-Moghaddam, Victoria Barkley, Mohammad Javan, Javad Mirnajafi-Zadeh, Akira Sumiyoshi, Mohammad Reza Raoufy

**Affiliations:** 1 Department of Physiology, Faculty of Medical Sciences, Tarbiat Modares University, Tehran, Iran; 2 Faculty of Electrical Engineering, Sharif University of Technology, Tehran, Iran; 3 School of ECE, College of Engineering, University of Tehran, Tehran, Iran; 4 Chronic Respiratory Diseases Research Center, National Research Institute of Tuberculosis and Lung Diseases (NRITLD), Shahid Beheshti University of Medical Sciences, Tehran, Iran; 5 Institute for Cognitive and Brain Sciences, Shahid Beheshti University, Tehran, Iran; 6 Department of Biomedical Engineering, Amirkabir University of Technology (Tehran Polytechnic), Tehran, Iran; 7 Krembil Research Institute, University Health Network, Toronto, Canada; 8 Department of Brain Sciences and Cognition, Cell Science Research Center, Royan Institute for Stem Cell Biology and Technology, ACECR, Tehran, Iran; 9 Institute for Brain Sciences and Cognition, Faculty of Medical Sciences, Tarbiat Modares University, Tehran, Iran; 10 Department of Functional Brain Imaging, IDAC, Tohoku University, Sendai, Japan; 11 National Institute on Drug Abuse Intramural Research Program, National Institutes of Health, Maryland, United States of America

**Keywords:** Anxiety, Asthma

## Abstract

Anxiety is prevalent in asthma, and is associated with disease severity and poor quality of life. However, no study to date provides direct experimental evidence for the effect of allergic inflammation on the structure and function of medial prefrontal cortex (mPFC) and amygdala, which are essential regions for modulating anxiety and its behavioral expression. We assessed the impact of ovalbumin (OVA)-induced allergic inflammation on the appearance of anxiety-like behavior, mPFC and amygdala volumes using MRI, and the mPFC-amygdala circuit activity in sensitized rats. Our findings exhibited that the OVA challenge in sensitized rats induced anxiety-like behavior, and led to more activated microglia and astrocytes in the mPFC and amygdala. We also found a negative correlation between anxiety-like behavior and amygdala volume. Moreover, OVA challenge in sensitized rats was associated with increases in mPFC and amygdala activity, elevation of amygdala delta-gamma coupling, and the enhancement of functional connectivity within mPFC-amygdala circuit – accompanied by an inverted direction of information transferred from the amygdala to the mPFC. We indicated that disrupting the dynamic interactions of the mPFC-amygdala circuit may contribute to the induction of anxiety-related behaviors with asthma. These findings could provide new insight to clarify the underlying mechanisms of allergic inflammation-induced psychiatric disorders related to asthma.

## Introduction

Asthma is a common chronic respiratory inflammation disease that is characterized by wheezing, shortness of breath, chest tightness and coughing. In addition to classical symptoms, psychiatric disorders, especially anxiety, are prevalent in asthmatic patients. Such disorders are associated with poor asthma outcomes^1^. A recent meta-analysis indicated that the pooled prevalence of anxiety disorders in youth with asthma is 22.7%, which is almost three times higher than in healthy youth^2^. The asthma-anxiety interaction leads to exacerbations in both psychological functioning and asthma, and adversely affects asthma control and quality of life^1^. This evidence highlights an association between asthma patients’ outcomes and the brain’s structural and functional alterations. Understanding such alterations may provide new pathophysiological insights into the characteristics of asthma, and hence may provide opportunities to develop novel interventions and therapeutic strategies.

A few studies, using imaging and histological methods, have identified an association between allergen exposure and alteration in brain areas linked to anxiety and emotional reactivity. In humans functional magnetic resonance imaging (fMRI) during the late phase of an asthma episode shows increased activity in the prefrontal cortex (PFC), which was suggested to be a possible reason for elevated anxiety and negative affect in patients with asthma^3^. Several rodent studies showed that exposing sensitized animals to an allergen might influence the immune and molecular profile of brain areas that involve emotion-relevant circuits and induce anxiety. Exposing sensitized rodents to ovalbumin (OVA) induced anxiety-like behavior, which was assessed in open field and elevated maze tests during the early phase of an allergic reaction^4,5^. OVA-sensitized rodents exhibited increased anxiety-like behavior, accompanied by the induction of TH2 cytokines and corticotropin-releasing factor in the PFC following OVA exposure^6^. Also, OVA challenge induced up-regulation of c-Fos protein in the amygdala and hypothalamus, along with increased anxiety-like behavior in sensitized mice^4^.

Findings from structural and functional investigations of the medial prefrontal cortex (mPFC) amygdala circuit highlight the top-down and bottom-up interactions between the mPFC and amygdala regions in representing states of high and low anxiety-related behaviors^7,8^. In humans, MRI and fMRI studies demonstrated a reduction of amygdala volume, and amygdala hyperactivity in anxiety disorders^9^. In response to an affective challenge, highly socially anxious individuals displayed increased cross-frequency coupling in the frontal electroencephalography signals^10^. In brief, cross frequency coupling is the interaction between the brain’s neuronal oscillations at different frequency bands. Cross-frequency analysis may be considered a neural code for facilitating information exchange across different brain regions^11^. This analysis is a useful method for assessing functional dynamics between ‘emotional’ and ‘cognitive’ brain networks^10^. For instance, delta-gamma coupling is demonstrated to be sensitive to external stimuli. Hence, delta-gamma coupling is suggested to be a possible mechanism for discriminating a good from a bad performance condition^12^. In addition, individual differences in anxiety are reflected in the intensity of mPFC-amygdala functional connectivity in resting-state fMRI^13^. In rodents, electrophysiological recordings revealed mPFC and amygdala hyperactivity associated with heightened anxiety-related behaviors^14,15^. Moreover, an optogenetic approach indicated that a causal relationship within mPFC-amygdala pathway activity modulates anxiety-like behavior in freely-moving mice^16^.

Although previous studies on the animal model of asthma described alterations of immune and molecular status in anxiety-related brain regions, we are unaware of any investigations focusing on the impact of asthma-induced allergic inflammation on the structure and function of the mPFC-amygdala circuit, which modulates anxiety-related behavior. Due to ethical and technical limits on human studies, assessing the effects of allergen exposure on the mPFC-amygdala circuit requires an animal model. Therefore, in sensitized rats, we evaluated the impact of OVA-induced allergic inflammation on the appearance of anxiety-like behavior, and investigated mPFC and amygdala volumes using MRI. Then, we focused on a freely-moving electrophysiological approach to uncover dynamic interactions between the mPFC and the amygdala in sensitized rats exposed to OVA.

## Methods

### Animals

Specific, pathogen-free, male Wistar rats weighing 80–100 g (4–5 weeks) were obtained from the Pastour Institute (Tehran, Iran), and were kept in standard animal research facilities with free access to food and water. The rats weighed between 250 and 300 g (10–11 weeks) at the time of the experiments. All protocols were performed in agreement with the guidelines of the NIH Guide for the Care and Use of Laboratory Animals (2011), and approved by the “Ethics Committee of Faculty of Medical Sciences, Tarbiat Modares University”.

### Experimental groups

**Figure 1:**
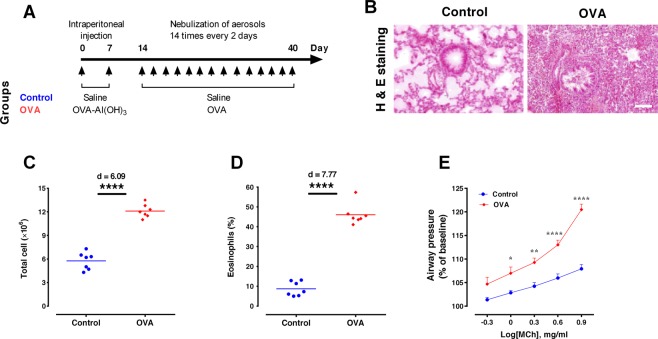
Allergen exposure increases airway hyper-responsiveness and lung inflammation. (**A**) Timeline of the study design. The rats received 2 intraperitoneal injections of saline or OVA-Al(OH)_3_ [1 mg OVA (Grade III; Sigma) and 100 mg Al(OH)_3_, in 1 ml saline] on day 0 and day 7, and 14 inhalation exposures (for 30 min, every 2 days, from day 14 to day 40) with aerosols of saline or OVA solution (2% wt/vol). (**B**) Hematoxylin and eosin (H&E) staining of lung sections to identify peribronchial inflammatory cell infiltration (Scale bar = 50 μm). (**C**) Airway hyper-responsiveness in response to increasing doses of methacholine. Data are presented as percent of baseline, expressed as mean ± SEM, and analyzed by repeated measures two-way ANOVA, with the Bonferroni post-hoc test, n = 7 per group. (**D**,**E**) Total BAL inflammatory cells and eosinophils, respectively. The horizontal bars represent mean values. Data were analyzed by t-test, n = 7 per group. *p < 0.05, ***p < 0.001 and ****p < 0.0001 compared to control. OVA, ovalbumin.

All animals received 2 intraperitoneal injections of saline or OVA-Al(OH)_3_ [1 mg OVA (Grade III; Sigma) and 100 mg Al(OH)_3_, in 1 ml saline] on days 0 and 7, and 14 aerosol inhalations (for 30 min every 2 days from day 14 to day 40) consisting of either saline or OVA solution (2% wt/vol) (Fig. 1A). We used unique animals in each test. However, some animals received more than one test comprising an MRI and the behavioral experiments.

### Assessment of allergic responses in the lung and brain

After 72 h from the last aerosol exposure, 14 rats (Control: n = 7 and OVA: n = 7) were used for assessment of airway hyper-responsiveness and bronchoalveolar lavage fluid (BALF) analysis. Animals were anaesthetized (1.5 g/kg urethane, intraperitoneally), tracheally cannulated, and ventilated with a volume-controlled ventilator (Harvard Apparatus, Holliston, MA) at a tidal volume of 1.0 ml/100 g and a rate of 70 breaths/min. Pancuronium bromide (0.2 mg/kg, intraperitoneally) was injected to prevent efforts against mechanical ventilation. For assessment of airway hyper-responsiveness, cumulative doses of MCh (0.5-1-2-4-8 mg/ml in saline) were inhaled for 60 s at 5-min intervals. The airway responsiveness was expressed as the percentage of airway pressure provoked by MCh to basal pressure^17,18^.

For BALF analysis, 3 ml of sterile physiological saline was slowly instilled and gently withdrawn into the syringe (3 times) through the endotracheal tube. Each sample was centrifuged, supernatant was discarded and the cells were washed thrice in RPMI Medium. Total cells and eosinophils were counted on BAL cytospin samples prepared by cytocentrifugation (cytospin 3, Shandon Instruments, Pittsburgh, PA), and stained with Giemsa stain^19^. After BALF collection, lungs were immediately removed and fixed in formalin 10%. Five-micrometer sections were obtained and stained with haematoxylin and eosin (H&E) to identify inflammatory cells that infiltrated the airway^18,19^.

For immunofluorescence staining, rats (Control: n = 3 and OVA: n = 3) were perfused transcardially with cold phosphate buffered saline (PBS) followed by 4% paraformaldehyde solution. Brains were extracted and post-fixed with paraformaldehyde overnight at 4 °C and cryoprotected for 48 h in 30% sucrose. Eight μm-thick coronal serial sections from the level of the mPFC and amygdala were cut and incubated overnight at 4 °C with primary antibody for anti-Iba1 (1:100, SC-98468), anti-GFAP (1:300, Z0334), anti-CD68 (1:200, ab955), anti-Olig2 (1:200, ab9610) and anti-NeuN (1:200, ab134014). Slides were washed with PBS and incubated with secondary antibody (goat anti-rabbit AlexaFluor®594; 1:1000, A-11036, goat anti-rabbit AlexaFluor®488; 1:1000, A-11008, goat anti-mouse AlexaFluor®488; 1:1000, A-11001) at room temperature for 1 h, followed by washing with PBS. Sections were counterstained with DAPI (blue) and imaged using fluorescence microscopy (Olympus BX51 TRF, USA).

For the quantification of either cell number or fluorescence intensity, at least four sections from the mPFC or amygdala from each animal were used. Digital images were captured from random, non-overlapping, consecutive microscopic fields from the mPFC and amygdala area using 200x magnification under an Olympus BX-51 microscope and DP72 camera (6 images/section, 4 sections/animals, 3 animals/group) and analyzed. A grid (50 μm × 50 μm) was randomly assigned on each image, and 7 squares were counted for Olig2^+^ and NeuN^+^ Cells. The number of total NeuN^+^ cells and Olig2^+^ cells were expressed as number of cells/mm^2^. GFAP, Iba1 and CD68 fluorescence intensity was determined by measuring the mean gray value of mPFC and amygdala areas. Each value was normalized to the mean of tissue background intensity and presented as the percent of control group. Fluorescence intensity and cell number were analyzed using Image J software.

### Animal behavioral tests

Twenty-four hours after the last aerosol exposure, open field and elevated zero maze tests were conducted on 26 animals (Control: n = 14 and OVA: n = 12). In order to exclude the effect of anesthesia and brain injury due to electrode implantation on the animals’ behavior, we used intact rats to consider those behavior changes that were directly induced by the experimental model of asthma. The open field test is an indicator of locomotor activity, and was carried out to assess total distance traveled. Animals were placed individually into a standard open field box and allowed to explore for 10 minutes. One rat in the control group in the open field test was excluded from analysis due to experimental error. For the elevated zero maze, animals were placed in the walled region and left undisturbed for 5 minutes. One rat in OVA group jumped off the elevated zero maze and was excluded from analysis. The time spent in the open sections and the number of open entries were measured as inverse indices of anxiety-like behavior.

### Brain MRI volumetry

All MRI data were acquired using a 3T Siemens Prisma (Siemens, Erlangen, Germany) scanner located in the National Brain Mapping Laboratory (NBML) (Tehran, Iran). Forty-eight hours after the last aerosol exposure, 31 rats (Control: n = 14 and OVA: n = 17) were anesthetized using ketamine-xylazine (100 and 10 mg/kg) and MRI procedures were performed with a 38-mm diameter birdcage coil that was designed to image the rat brain. T2-weighted images were obtained using turbo spin echo (TSE) sequence with the following parameters: TR = 4570 ms, TE = 90 ms, FOV = 64 × 64 mm^2^, voxel size = 0.2 × 0.2 × 0.8 mm, number of slices = 39 and slice thickness = 0.8 mm. Images were processed with Advanced Normalization Tools (ANTs) software package^20^ according to methods previously described^21^ as follows: (i) images were exported in DICOM format and converted to NIfTI format using “dcm2niix” software; (ii) images were manually rotated and translated, such that the origin of the coordinates occupied the midpoint of the anterior commissure to roughly match the standard reference space; (iii) a single reference image was manually skull-stripped using “ITK-SNAP” software to create the brain mask; (iv) other subject images were registered to the reference image and skull-stripped; (v) image non-uniformity was corrected, and image intensity was normalized; (vi) a minimum deformation template (MDT) was constructed with the following SyN parameters: gradient step size = 0.1 voxels, update field variance = 3 voxels, and total field variance = 0.5 voxels; (vii) the obtained MDT was manually skull-stripped and registered to the common atlas space^22^ which has 138 ROIs by combining several rat brain MRI atlases in the literature^23–25^; (viii) each ROI volume was computed, divided by the total brain volume to account for the brain size differences, and analyzed.

### Electrophysiology and signal processing

Sixteen animals (Control: n = 8 and OVA: n = 8) were anesthetized with intraperitoneal injections of ketamine-xylazine anesthesia (100 and 10 mg/kg) and mounted on a stereotaxic apparatus (Narishige, Japan) in a flat skull position. For local anesthesia, 0.5 ml of lidocaine chlorhydrate 2% was subcutaneously injected in the scalp before surgery. The core body temperature was maintained at 37 °C by a heating pad throughout the surgery. After exposing the skull, stainless-steel recording electrodes (127 µm in diameter, A.M. system Inc., USA) were implanted unilaterally into the prelimbic cortex of mPFC (AP: +3.2 mm; L: 0.6 mm; DV: −3.6 mm) and the basolateral amygdala (BLA) (AP: −2.5 mm, L: 5 mm, DV: −7.4 mm)^26^. A stainless-steel screw placed at the right parietal bone was used as reference. The scalp skin was treated with antibiotics (tetracycline). Small pins were attached to the electrodes and then inserted to a small socket. Dental acrylic cement was used for the whole structure fixation on the skull.

Seven days after surgery (24 hours after the last aerosol exposure), local field potentials (LFPs) were recorded for 5 minutes in the animal’s home cage, and 30 s periods of awake immobility were collected for signal processing. For this purpose, the socket, fixed to the animal’s head, was connected to a miniature buffer headstage with high-input impedance (BIODAC-A, TRITA WaveGram Co., Tehran, Iran), via cables to a main AC coupled amplifier (1000 amplification) and to the recording system (BIODAC-ESR18622, TRITA WaveGram Co., Tehran, Iran). Spontaneous LFPs of mPFC and amygdala were simultaneously recorded by two distinct channels, low-pass filtered <500 Hz, digitized at 1 KHz, and stored for offline analyses with custom-written MATLAB routines (The Mathworks, Inc.). A video tracker was used for recording the movement of animals in order to detect awake immobility periods.

For histological verification of electrodes position, rats were deeply anaesthetized with an intraperitoneal injection of urethane (1.2 g/Kg) and perfused transcardially with PBS followed by 4% paraformaldehyde solution. Brains were extracted and post-fixed with paraformaldehyde overnight. Coronal sections were cut (50 μm) and mounted on glass slides. Electrodes’ positions were verified by light microscopy.

LFP data from 30 s periods of awake immobility were analyzed offline in MATLAB (The Mathworks, Inc.) using built-in and custom-written routines. These LFP time series were divided into 3 segments of length 10 s each. Analyses were performed on these segments, and the average of obtained data was considered for each animal. Power spectral density (PSD) was calculated by means of Welch’s periodogram (built-in MATLAB pwelch function; 90% overlapping 6 s Hamming windows). Coherence spectra were computed for mPFC and amygdala using magnitude-squared coherence (built-in MATLAB mscohere function; 90% overlapping 6 s Hamming windows). The cross-correlation lag analysis for the delta (<4 Hz) and theta (4–12 Hz) filtered signal was calculated using the xcorr function, with the “coeff” option to normalize values between −1 and 1.

Phase-amplitude cross-frequency coupling was computed using a previously described framework^27^. We analyzed the coupling between the phase of the delta (<4 Hz) and theta (4–12 Hz) bands (0.5 Hz increments), and the amplitude of the gamma band between 30 and 120 Hz (1 Hz increments). The delta/theta phases were binned into eighteen, 20° intervals, and the corresponding gamma amplitude was averaged for each phase bin. This process gives rise to a phase-amplitude profile. The phase-amplitude modulation index (MI) measures the divergence of the empirical phase-amplitude profile from the uniform distribution, which characterizes the lack of coupling. The comodulation maps were obtained by computing the MI between multiple band-filtered frequency pairs and expressing the results in a 2D pseudocolor plot.

### Statistical analysis

GraphPad Prism (GraphPad Software, San Diego, CA) was used for statistical analysis. The normality of the data distribution within each parameter was calculated by Kolmogorov-Smirnov test. Differences between control and OVA groups were analyzed by Mann–Whitney test for immunofluorescence data and by t-test for BALF, behavioral, MRI volumetry and local field potentials analysis. The Pearson correlation coefficient was used to evaluate the correlation between animals’ behavioral test performance and brain MRI volumetry. Changes in airway pressure for groups were compared using a two-way ANOVA, with Bonferroni post-hoc corrections where applicable. P-values less than 0.05 were considered statistically significant. Effect size was calculated using Cohen’s *d* by MATLAB software.

## Results

### Allergic responses in the lung and brain

Histopathological findings revealed that OVA challenge induced an intense peribronchovascular inflammatory infiltrate in the lungs of sensitized rats (Fig. 1B). In the BALF, the level of total inflammatory cells and eosinophils were higher in the OVA group (p < 0.001, d = 6.09; p < 0.001, d = 7.77, respectively) (Fig. 1C,D). OVA challenge also augmented airway responsiveness to cumulative doses of MCh in sensitized rats (p < 0.001) (Fig. 1E).

**Figure 2:**
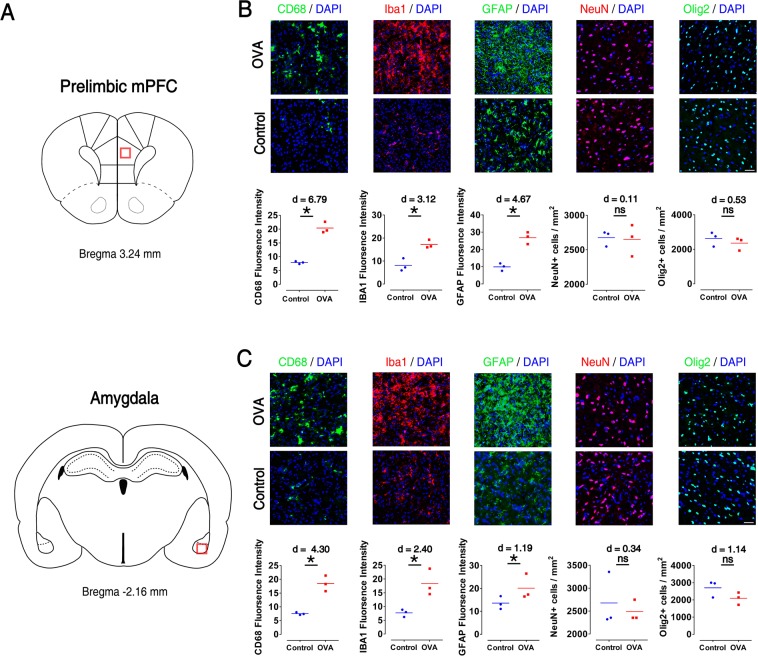
Microglia and astrocytes were activated and oligodendrocytes were reduced in prelimbic mPFC and basolateral amygdala of allergen-exposured rats. (**A**) Displays schematics indicate regions of immunostaining in the brain. (**B**,**C**) Representative immunoreactivity images of microgelia (CD68 and Iba1), astrocyte (GFAP), neuronal survival (NeuN) and oligodendrocyte (Olig2) in prelimbic mPFC (**B**) and basolateral amygdala (**C**) as well as quantitative assessment on each test (lower panels). Scale bar represents 50 μm. Data were analyzed by Mann–Whitney test, n = 3 per group. *p < 0.05 compared to control. OVA, ovalbumin; mPFC, medical prefrontal cortex.

As shown in Fig. 2, immunofluorescence staining of brain sections revealed increased microglia reactivity of mPFC (Iba1+ microglia: p < 0.05, d = 3.12; CD68 expression: p < 0.05, d = 6.79) and amygdala (Iba1+ microglia: p < 0.05, d = 2.40; CD68 expression: p < 0.05, d = 4.30) in the OVA group. Also, GFAP, as a marker of astrocytes, was augmented in the mPFC and amygdala of OVA animals compared to controls (mPFC: p < 0.05, d = 4.67; amygdala: p < 0.05, d = 1.19). OVA exposure did not significantly alter the number of NeuN^+^ (a marker of surviving neurons) and Olig2^+^ (an oligodendrocyte lineage marker) cells in mPFC and amygdala of sensitized rats. However, a large effect size reduction in amygdala Olig2^+^ cells was observed in OVA group compared to control animals (p = 0.1, d = 1.14).

### Allergen induces anxiety-like behavior

**Figure 3:**
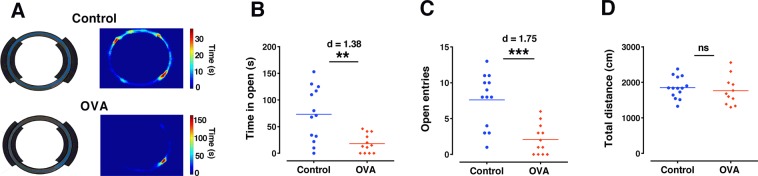
Allergen exposure induces anxiety-like behavior. (**A**) Representative animal track in the elevated zero maze for a control (upper panels) and an OVA (lower panels) rat. (**B**,**C**) Time spent in open sections and number of entries to the open sections in the elevated zero maze, which inversely related to anxiety-like behavior respectively (Control: n = 14, OVA: n = 11). (**D**) Total distance traveled (as an indicator of locomotor activity) in the open field test (Control: n = 13, OVA: n = 12). The horizontal bars represent mean values. Data were analyzed by t-test. **p < 0.01, ***p < 0.001 compared to control. ns, not significant; OVA, ovalbumin.

A representative animal track from the control and OVA groups are shown in Fig. 3A. OVA challenge in sensitized rats significantly decreased time spent in the open sections (p < 0.01, d = 1.38) and the number of open-section entries (p < 0.001, d = 1.75) in the elevated zero maze test, which indicates an increase in anxiety-like behavior in the OVA group (Fig. 3B,C). That there was no difference in the total distance traveled within the open field test rules out a possible influence of reduced locomotion due to allergen exposure (Fig. 3D).

### Anxiety-like behavior is negatively correlated with amygdala volume

**Figure 4:**
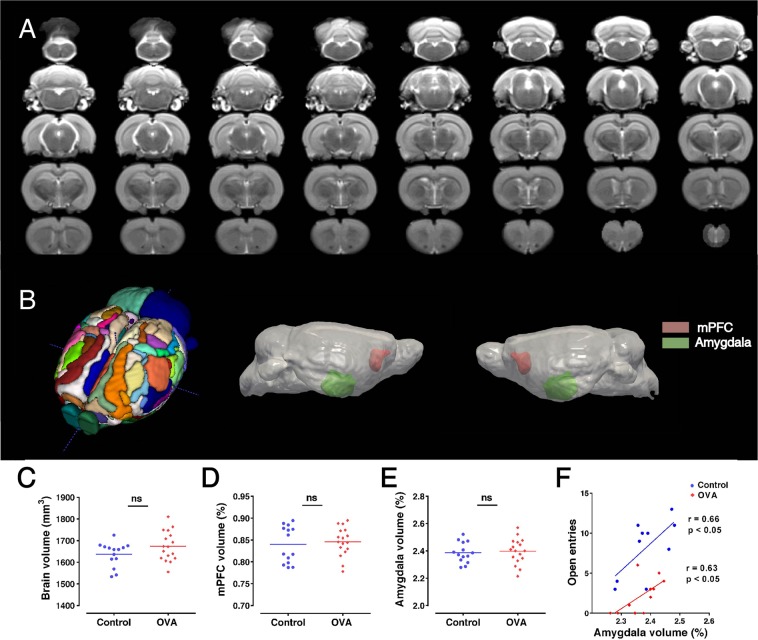
Anxiety-like behavior is negatively correlated with amygdala volume. (**A**) Representative T2-weighted coronal MRI slices, which were registered to template. (**B**) Displays three-dimensional rendering views of atlas (left panel), lateral representative of brain (middle and right panel). Regional selections of mPFC and amygdala have shown red and green, respectively. (**C**–**E**) MRI volumetric measurements of whole brain, mPFC and amygdala, respectively. The horizontal bars represent mean values (as percent of brain volume). Data were analyzed by t-test (Control: n = 14, OVA: n = 17). (**F**) The number of entries to the open sections in elevated zero maze (inversely related to anxiety) was positively correlated with amygdala volume. ns, not significant; OVA, ovalbumin; mPFC, medical prefrontal cortex.

Figure 4 demonstrates representative T2-weighted coronal MRI slices that are registered to a template (Fig. 4A), and MRI volumetric measurements of whole brain, mPFC and amygdala (Fig. 4B–D). OVA challenge in sensitized rats had no significant effect on the whole brain volume (mm^3^) and the percent of mPFC and amygdala volumes. However, the number of entries to the open sections in elevated zero maze test (inversely related to anxiety) was positively correlated with amygdala volume (control group: r = 0.66, p < 0.05; OVA group: r = 0.63, p < 0.05) (Fig. 4E).

### Allergen enhances delta and theta activity in mPFC and amygdala

**Figure 5:**
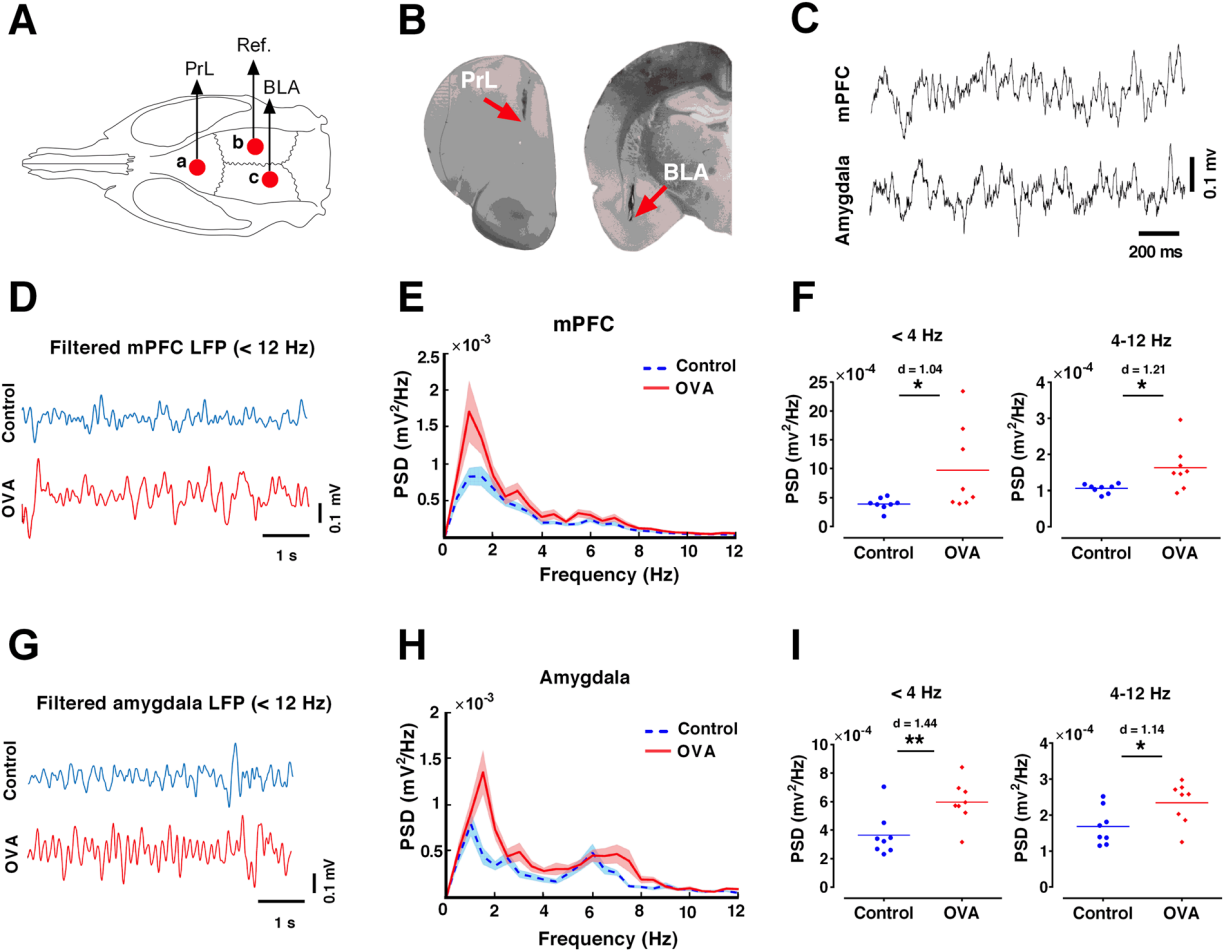
Allergen exposure increases delta and theta activity in mPFC and amygdala. (**A**) Schematic representation of electrode implantation sites. (**B**) Example histology of electrodes placed in PrL and BLA. (**C**) Representative raw traces of simultaneous LFP recordings from the mPFC and amygdala. (**D**,**G**) Examples of mPFC and amygdala LFP traces filtered at delta and theta frequencies (<12 Hz). (**E**,**H**) Averaged power spectra of LFPs in the mPFC and amygdala. Shaded area indicates standard errors. (**F**,**I**) OVA exposure increases delta (<4 Hz) and theta (4–12 Hz) power spectral density of the mPFC and amygdala LFPs. The horizontal bars represent mean values. Data were analyzed by t-test, n = 8 per group. *p < 0.05, **p < 0.01 compared to control. OVA, ovalbumin; mPFC, medical prefrontal cortex; PrL, prelimbic; BLA, basolateral amygdala; LFP, local field potential.

We used an extracellular recording technique in awake rats to measure LFPs in the mPFC and amygdala (Fig. 5A–C). Figure 5D,G exhibits large voltage oscillations in the delta and theta frequency range (<12 Hz) in the mPFC and amygdala of Control and OVA groups. Spectral analysis of mPFC and amygdala revealed a higher delta and theta power in the OVA group compared to the control group, with a prominent power peak at delta frequency (Fig. 5E,H). OVA challenge in sensitized rats significantly increased delta (<4 Hz) and theta (4–12 Hz) activity in mPFC (p < 0.05, d = 1.04; p < 0.05, d = 1.21, respectively) and amygdala (p < 0.01, d = 1.44; p < 0.05, d = 1.14, respectively) (Fig. 5F,I).

### Allergen disrupts mPFC-amygdala circuit

**Figure 6:**
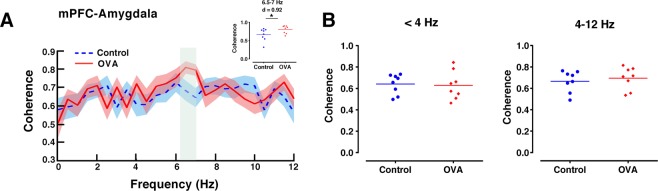
Coherence between mPFC and amygdala is increased at theta frequency in allergen-exposured rats. (**A**) Spectral coherence between simultaneously recorded mPFC and amygdala LFPs. Shaded area indicates standard errors. The gray area indicates significant differences between OVA and control rats. Inserted panel shows significant differences between OVA and control rats at 6.5–7 Hz frequencies. (**B**) Mean mPFC-amygdala coherence at delta (<4 Hz, left) and theta (4–12 Hz, right) frequencies. The horizontal bars represent mean values. Data were analyzed by t-test, n = 8 per group. *p < 0.05 compared to control. OVA, ovalbumin; mPFC, medical prefrontal cortex; LFP, local field potential.

Since theta-frequency oscillations have important impact in long-range synchrony within the mPFC-amygdala circuit during anxiety^8^, we studied whether such synchrony was altered by allergen presence. Coherence analysis of the signals obtained simultaneously from the mPFC and amygdala demonstrated that OVA challenge in sensitized rats significantly enhanced theta (6.5–7 Hz) frequency coupling between these brain regions, while delta-frequency coherence was not significant (Fig. 6A,B).

**Figure 7:**
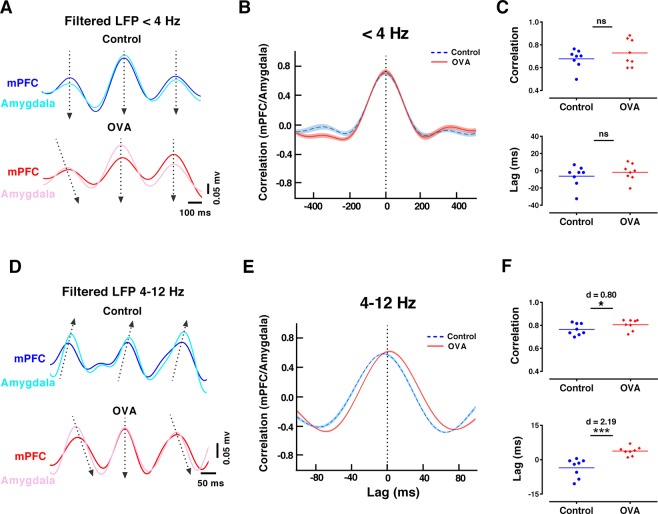
Allergen exposure enhances synchrony and inverses directionality of theta activity in mPFC-amygdala circuit. (**A**,**D**) Examples of mPFC and amygdala delta (<4 Hz) and theta (4–12 Hz) filtered signals, illustrating the cross-correlation lag analysis in the control (upper) and OVA (lower). Arrows are drawn from the leading area to the lagging area. (**B**,**E**) Mean waveform correlation for the delta and theta-filtered signals in the mPFC-amygdala circuit. Shaded area indicates standard errors. (**C**,**F**) Mean mPFC-amygdala correlation and lag at delta (<4 Hz) and theta (4–12 Hz) frequencies. OVA exposure increases correlation for theta oscillation between mPFC and amygdala with a delay in the mPFC. The horizontal bars represent mean values. Data were analyzed by t-test, n = 8 per group. *p < 0.05, ***p < 0.001 compared to control. OVA, ovalbumin; mPFC, medical prefrontal cortex; LFP, local field potential.

To further explore the dynamics and directionality of the mPFC-amygdala circuit, we used cross-correlation analysis to calculate mean correlation and lag time of delta and theta oscillations between LFPs recorded from the mPFC and amygdala. The mean mPFC-amygdala correlation and lag time for the delta filtered signals (<4 Hz) in two groups was similar (Fig. 7A–C). Closer inspection of the filtered signal in control group indicated that theta oscillations (4–12 Hz) in mPFC preceded the activity recorded in the amygdala for multiple cycles (Fig. 7D,E). However, OVA challenge in sensitized rats increased correlation (p < 0.05, d = 0.80) and inverted the direction of theta activity from the amygdala to the mPFC with a delay of around 3.75 ± 0.67 ms (p < 0.001, d = 2.19) (Fig. 7D–F).

### Allergen increases amygdala delta-gamma coupling

**Figure 8:**
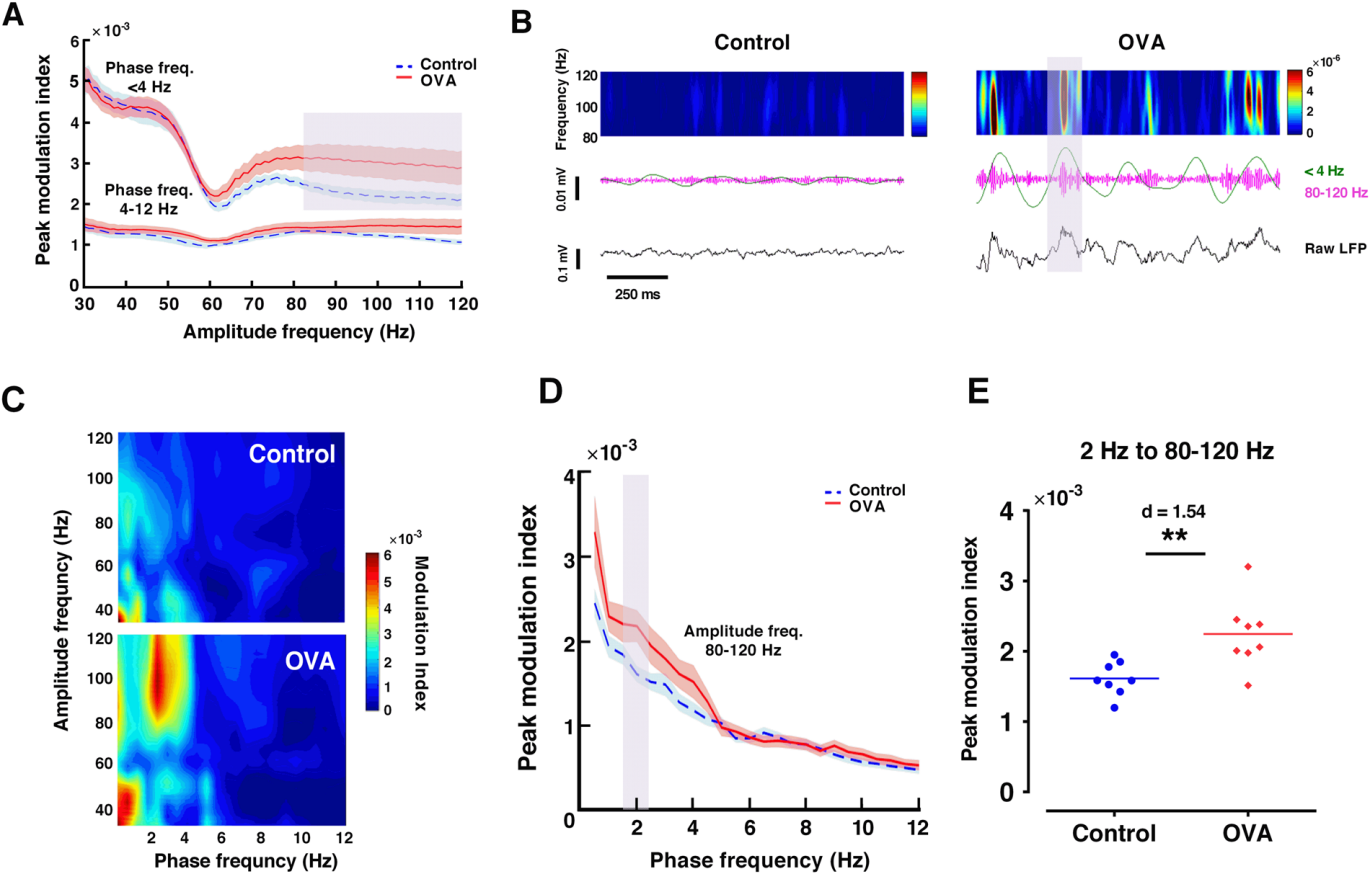
Allergen exposure increases phase-amplitude coupling of delta and gamma oscillations in amygdala. (**A**) Peak modulation index across amplitude frequencies computed for delta (<4 Hz) and theta phase (4–12 Hz) in amygdala. Shaded area indicates standard errors. The gray area indicates significant differences between OVA and control rats. Delta modulates 80–120 gamma activity in OVA-exposured rats. (**B**) Spectrograms (color plots) of amygdala LFP (black, lower panels) and its delta (<4 Hz, green) and gamma (80–120 Hz, pink) filtered versions (middle panels) in control (left panels) and OVA (right panels) rats. Gray area indicates a representative high-amplitude gamma event occurring at the crest of delta phase. (**C**) Phase-amplitude comodugram of representative amygdala LFP recordings. (**D**) Peak modulation index across phase frequencies computed for gamma amplitude (80–120 Hz) in amygdala. Shaded area indicates standard errors. The gray area indicates significant differences between OVA and control rats. (**E**) Delta modulation of gamma increases for 2 Hz in OVA-exposured rats. The horizontal bars represent mean values. Data were analyzed by t-test, n = 8 per group. **p < 0.01 compared to control. OVA, ovalbumin; LFP, local field potential.

Given the importance of coupling between the phase of low frequency oscillations and the amplitude of high frequency oscillations during episodes of fear and anxiety^28^, we examined whether exposing sensitized rats to OVA increases delta/theta-gamma coupling in the mPFC and amygdala. Peak comodulation frequencies for amygdala gamma oscillations revealed that delta (<4 Hz) strongly modulates fast gamma (80–120 Hz) activity in OVA group (Fig. 8A). Peak modulation index across phase frequencies indicated that OVA challenge in sensitized rats significantly augmented amygdala delta-fast gamma coupling when the instantaneous frequency of delta was 2 Hz (p < 0.01, d = 1.54) (Fig. 8D,E). There was no significant difference in mPFC delta/theta-gamma coupling between groups (data not shown).

## Discussion

In patients with asthma, psychiatric disorders, especially anxiety, have significant impact on public health: more visits to health care providers, more emergency room admissions, and more frequent withdrawal from participation in regular activities^1,29^. Although comorbidity between anxiety and asthma has attracted attention over the past several decades, controversies exist concerning the direction of causality between these two conditions. A meta-analysis indicated that allergy and atopic conditions strongly increase the risk of subsequent psychological dysfunction, and allergy-related inflammatory responses are suggested as a main pathway linking asthma and behavioral changes^2,30^. However, to the best our knowledge, there is no study that provides direct experimental evidence for the effect of allergic inflammation on structural and functional alterations in brain regions related to anxiety behaviors. We revealed for the first time that allergen exposure in sensitized rats was associated with changes in the dynamic interactions of mPFC-amygdala circuit, which is an important functional network in modulating the expression of anxiety-related behavior.

Asthma is associated with inflammation of the airways; and some of asthma’s manifestations can be a result of a reciprocal interaction between the immune and nervous system, known as “immune to brain communication”^31^. There are three essential pathways, including circumventricular organs (CVOs) via the cerebral endothelium or the vagus nerve, that have been proposed for the central nervous system interaction with the immune system^32^. Firstly CVOs have an incomplete blood brain barrier (BBB), which allow free diffusion immune mediators into the brain^33^. Cytokine receptor productions of the endothelial cells in the brain parenchyma were considered as a second pathway. The immune system releases mediators into the brain, which can interact with glia or neurons to stimulate neuronal activation^34^. Moreover, the vagus nerve can transmit inflammatory signals to the nucleus of solitary tract (NTS) in the brain, which projects to many other brain regions^31,35^. Together, these communication mechanisms facilitate activation of different brain nuclei leading to metabolic and behavioral changes associated with inflammation^32^. Moreover, there is evidence that allergen-induced, peripheral inflammation triggers increases in the expression of TH2 cytokines together with microglia and astrocyte activation in various regions of the central nervous system, and this might contribute to emotional disorders associated with asthma^6,36^. In this context, our study also exhibited that the OVA challenge in sensitized rats resulted in an influx of inflammatory cells into the lungs and led to more activated microglia and astrocytes in the mPFC and amygdala. A recent study indicated that an infusion of inflammatory cytokine in the hypothalamus’ paraventricular nucleus (PVN) increased GFAP expression with associated enhanced anxiety-like behaviors. It appears that an abnormally augmented GABA release from reactive astrocytes in the hypothalamus may be important in pathophysiology of anxiety disorders^37^. Furthermore, evidence also suggested that anxiety-like behavior is accompanied by microglial activation, that is, an enhanced number of Iba1+ and CD68+ cells^38,39^. Furthermore, our findings suggested that OVA exposure reduced oligodendrocytes in the amygdala with a large effect size, although this change was not significant due to our small sample size. This observation is in accord with the reports that state that systemic inflammation diminishes proliferation of oligodendrocyte progenitor cells in the adult hippocampus^40,41^.

The amygdala is connected to widespread, emotional-related brain regions (such as the mPFC, hippocampus, anterior cingulate cortex, etc.), and is considered an essential neural hub for the modulation of anxiety behaviors^42^. It is believed that anxiety responses are inducted by the information conveyed to these brain regions from the amygdala^42^. Several human and animal studies used structural neuroimaging to explore an association between anxiety behaviors and amygdala volume, but the sum of their results were inconsistent. There is more consistent evidence for a negative directional association between these two variables in animal studies, as well as in a few human reports^43–46^. Although we found no significant difference in amygdala volume between sensitized rats exposed to OVA and controls, which is consistent with previous studies, our results showed a negative correlation between the level of anxiety-like behavior and amygdala volume.

On the other hand, we observed that OVA challenge in sensitized rats induced anxiety-like behavior and also increased amygdala activity, as observed in enhanced delta and theta power. In agreement with our findings, rodent studies reported an association between amygdala hyperexcitability and anxiety-like behaviors^8,14^. Investigations on humans displayed amygdala hyperactivity with most types of anxiety behaviors^9,47^. An important question raised by these results is why the direction of correlation between anxiety-like behavior and amygdala volume appears negative, while increased anxiety appears to be associated with enhancement of amygdala activity. It seems that persistent amygdala hyperactivation and perturbations in its afferent inputs may lead to glutamatergic excitotoxicity process, and a subsequent decrease in amygdala volume is associated with increased anxiety-related behaviors^48^. However, the precise mechanisms for this observed association are yet unknown.

Given our findings of amygdala delta and theta power enhancements in an experimental model of asthma, we asked whether OVA challenge in sensitized rats could also increase the amygdala’s delta/theta-gamma coupling. We found that fast gamma activity (80–120 Hz) was strongly coupled with oscillations in delta frequencies in sensitized rats exposed to OVA. Phase-amplitude coupling denotes a synchronization of slow oscillations phase with amplitude of fast frequency waves and is considered as an essential mechanism to modulate emotional behavior^28^. Human studies suggested that in an anxiogenic situation, individuals with higher levels of anxiety-related behaviors tend to show increased phase-amplitude coupling in local cortical regions^10,49^. In line with our findings, a rodent study observed heightened theta-fast gamma coupling of the amygdala during fear expression, and suggested that amygdala fast gamma dynamically switched between states of fear and safety^28^. Although all neurophysiological concepts of this phenomenon remain unexplored, it is mostly acknowledged that slow oscillations have a major role in the timing of neuronal activities and may coordinate functions of the brain, such as emotional behaviors^50^.

In addition to the amygdala, the functional role of mPFC is also implicated in the modulation of anxiety-related behaviors. Electrophysiological studies in rodents exhibited that the mPFC is capable of generating neural signals, especially at theta band, which is correlated with heightened anxiety-like behavior^8,15^. In the open field test and elevated plus maze, theta power increase in mPFC was observed in the threatening zones, and suggested that increased mPFC theta wave activity contributed to inhibition of exploratory behavior^51,52^. Along this line, our findings showed that OVA challenge in sensitized rats enhanced delta and theta power in mPFC.

There is evidence that a decline of astrocytes in the mPFC attenuates power (but not cross-frequency coupling) of delta, alpha and gamma oscillations and causes cognitive impairment^53^. It seems that astrocytes regulate rhythmic firing in neurons^54^, and loss of astrocyte function impairs neuronal oscillations and cognitive performance^55^. Our study exhibits that astrocyte augmentation is likely to intensify power of delta and theta in mPFC and amygdala. This observation, in concert with previous studies, provide some evidence that a correlation might be between number of astrocytes and neuronal oscillations, and these cells seem to have essential role for brain functions, including behavioral regulation.

Although the anxiolytic effects of mPFC inactivation were previously described, in contrast, some investigations have shown anxiogenic effects of pharmacological inactivation of the mPFC^56,57^. It appears that the mPFC has a dual function in the expression of anxiety-related behaviors. The mPFC shares reciprocal projections with the widespread emotion-related brain regions, including the amygdala, and plays an essential role in the assessing external conditions to appropriately promote or suppress a wide range of anxiety and emotional behaviors^16^. In both humans and animals, ongoing mPFC-amygdala communication has proven to be critical in the sustained experience of anxiety or safety. The ventromedial PFC (vmPFC) has been implicated in the emotional control via down-regulation of amygdala activity^58^. During the safety period in humans, vmPFC activity was positively coupled with activity in the amygdala, while decreased vmPFC-amygdala coupling was reported during anticipatory anxiety^59^. In this context, there is evidence that the administration of inflammatory stimuli induced symptoms of anxiety in association with reduced functional connectivity between the vmPFC and amygdala^60^. In contrast, the dorsomedial prefrontal cortex (dmPFC) has a crucial role in the expression of anxiety behaviors and maintains defensive readiness during anxiety by enhancing the synchronized activity with anxiety-related brain regions, especially amygdala^59^. During anticipatory anxiety, dmPFC activity was positively coupled with activity in the amygdala, and a positive correlation between this coupling and the level of state anxiety was reported in humans^13,59^. The dmPFC is identified as a functional homologue of prelimbic mPFC in rodents by sustaining anxiety^61^, potentially priming the amygdala for producing appropriate defensive behaviors. A study demonstrated a positive correlation between mPFC-BLA synchrony and center-avoidance in the open-field test, and exhibited the elevation of theta oscillations in the mPFC-BLA circuit caused by anxiogenic stimuli in fear conditioning test^8^. Along this line, our cross-correlation and coherence analysis on LFP signals, from electrodes implanted in the prelimbic mPFC and BLA regions, revealed that OVA challenge in sensitized rats augmented anxiety-like behavior and also enhanced functional connectivity in the theta band within the mPFC-AMG circuit. In addition, the interaction dynamics’ directional change within mPFC-amygdala circuit is noted as important in the behavioral manifestations of anxiety and safety^8,16^. Therefore, we calculated the lag time of oscillations between LFPs recorded from the mPFC and amygdala to further explore the dynamics and directionality of the mPFC-amygdala circuit. Our results showed that theta oscillations in the mPFC preceded the activity recorded in the amygdala in control animals. It seems that the observed directionality is a consequence of safety-related directional theta information transferred from the mPFC to the amygdala, as described in previous studies^8,28^. In contrast, OVA challenge in sensitized rats was accompanied by theta activity’s directional inversion, from the amygdala to the mPFC. These results are consistent with evidence that indicates a causal role for amygdala projections to the mPFC in the experience of anxiety-related behaviors. Optogenetic approaches in behaving mice demonstrated that activation of BLA inputs to the mPFC induced anxiogenic effects in subjects using the elevated plus maze and open field test^16^. It also suggested that ascending projections from the BLA increase the activity of prelimbic mPFC leading to elevated fear responses^62^, and anxiogenic stimuli-induced mPFC entrainment to theta inputs from the BLA in fear conditioning test^8^.

Respiratory parameters can also be affected by higher-order and emotional influences^63^. It is well known that anxiety and arousing stimuli can provoke lung function impairment in asthma, which is associated with disease severity^64^. Since the amygdala is recognized as a key structure in processing aspects of anxiety-induced respiratory effects^63^, it appears that allergic inflammation induced by asthma may disrupt the top-down and bottom-up interactions between the amygdala and respiratory control network. This in turn results in inappropriate respiratory response to anxiety and arousing stimuli^63–66^. However, we did not examine the effect of airway inflammation on the interaction between respiratory dynamics and the activity of anxiety-related brain regions. This issue remains to be investigated in future studies.

In conclusion, we identified the direct effect of allergen-induced airway inflammation on the manifestation of anxiety behavior, and the structure and function of mPFC-amygdala circuit, which are important anxiety-related brain regions. Allergen-induced brain inflammation increased the mPFC and amygdala activity, and enhanced the functional connectivity within the mPFC-amygdala circuit with the direction of information inverted and transfer from the amygdala to the mPFC. Overall, although anxiety-like behavior was not assessed in the animals that provided electrophysiological data, our findings suggest a possible involvement of the mPFC and amygdala for the initiation and promotion of anxiety-related behaviors in asthma. Despite the limitations of our experimental model, such as the lack of a direct correlation between cellular and molecular changes, volumetric measurements and the activity of mPFC-amygdala circuit, our results could provide new insight to explain the underlying mechanisms of allergic inflammation-induced, neurobehavioral disorders in asthma. Further study is needed to identify the processes that explain anxiety-related behaviors in asthma, particularly role of hormonal alterations, such as the CRH-ACTH-cortisol system – this can be another piece of puzzle. Finally, these findings may contribute to a novel understanding of the pathophysiology of asthma. Hence, the current findings may lay the groundwork for developing new interventions for improving the outcome of patients with allergic asthma.
